# Clinical observation of tear film breakup time with a novel smartphone-attachable technology

**DOI:** 10.1186/s12886-023-02932-2

**Published:** 2023-05-10

**Authors:** Xiaoran Zhang, Jiawei Shen, Zhen Kang, Andrew Chen, Qian Cao, Chunyan Xue

**Affiliations:** 1 Department of Ophthalmology, Nanjing Jinling Hospital, Affiliated Hospital of Medical School, Nanjing University, Nanjing, Jiangsu 210002 China; 2 Department of Biology, Stony Brook University, New York, USA

**Keywords:** Lens attachment for smartphones, Slit-lamp microscope, Tear film breakup time

## Abstract

**Purpose:**

To demonstrate the practicability of a portable instrument in assessing tear film breakup time (TFBUT): a lens attachment for smartphones (LAS).

**Methods:**

By applying LAS in combination with the iPhone 12 pro, and a recordable slit-lamp microscope, we obtained TFBUT videos from 58 volunteers. The comparison between the conventional slit-lamp microscope and LAS by an experienced ophthalmologist. Moreover, we also invited an ophthalmic postgraduate student and an undergraduate student with no clinical experience to assess TFBUT in those videos. The inter-observer reliability was assessed using intraclass correlation coefficients (ICC).

**Results:**

The TFBUT of 116 eyes in total was recorded. Reliability indexes were adequate. The Spearman’s correlation analysis and the intraclass correlation coefficient suggested a strong correlation between the 2 modalities (Right eye: Spearman’s *r* = 0.929, 95% confidence interval (CI) = 0.847–0.963, ICC = 0.978, *p* < 0.001; Left eye: Spearman’s *r* = 0.931, 95% CI = 0.866–0.964, ICC = 0.985, *p* < 0.001;). Between instruments, the majority of TFBUT measurements showed good agreement on Bland Altman plot. A high concordance was observed in TFBUT, when assessed by an ophthalmologist and an ophthalmic postgraduate student (Left eye: LAS ICC = 0.951, *p* < 0.001; Left eye: slit-lamp microscope ICC = 0.944, *p* < 0.001).

**Conclusions:**

Compared with the conventional slit-lamp microscope, the LAS has sufficient validity for evaluating TFBUT in clinics or at home.

## Introduction

Dry eye disease (DED) is one of the most common ophthalmic diseases and reasons for seeking medical care [1–3]. The incidence of DED ranges from 5 to 50% [4], and is expected to rise along with various factors, such as aging, stress, and prolonged visual display terminal (VDT) use [1, 2, 5, 6]. In the continuous rising incidence context with DED, over the past decades, different organizations have defined a variety of concepts and diagnostic criteria for DED. In 2017, the most comprehensive DED definition was made from the TFOS DEWS II Report, which was the fruit of a seminar involving experts from various walks of DED [5]. The Asia Dry Eye Society also came up with a novel definition in the same year [6, 7]. Although several discrepancies exist among the standard for diagnosis, all of these definitions highlight that tear film instability is a critical factor in diagnosis of DED [1, 7–9].

Evaluation of tear film stability reveals important clues to ophthalmologists for clinical assessment and diagnosis, since a balanced tear film is a basis of anterior segment health. Ever since the invention of fluorescein based TFBUT by Norn in 1969 [10], various techniques, including both noninvasive and invasive TFBUT assessment methods, have been employed to assess tear film stability [4]. Among all those methods, fluorescence tear film breakup time evaluated by a slit-lamp microscope remains to be the most widely used one for its convenience and efficacy [11].

However, the conventional slit-lamp microscope evaluation is generally restricted by its necessity and requirement of both instrument and eye doctors. This restriction might limit the accessibility of this exam among certain patients, such as bedbound patients, poorly cooperative patients due to age or mental status, or people in isolation, etc. [12–15]. Even though with the availability of potable slit-lamp microscopes, documentation through photos or videos remains to be an issue. Furthermore, lacking appropriate medical equipment often hampers the timely diagnosis of certain eye diseases, particularly in underserved areas. DED sometimes may be associated with decreased vision acuity, poor job efficiency, and lower life quality without being diagnosed and treated in a timely manner [6]. Therefore, we intended to exploit a self-sifting device for DED diagnosis that can overcome the restriction of the conventional slit-lamp method.

Luckily, we have found a novel attachment called "lens attachment for smartphones". This product can change the light source from the smartphone into the one required by the ophthalmology diagnosis and can also provide the blue light needed for the ocular surface examination. When it is paired with any smartphone, it is able to provide a new method for ocular surface scanning and assessment with lower cost and easier accessibility compared with conventional slit-lamp examination.

In this study, we are presenting a novel method for TFBUT examination using a smartphone and LAS. We are also here reporting a study in which the TFBUT of volunteers was assessed using both the LAS and conventional silt-lamp microscope. Lastly, we used the intra-class correlation of the two-way random model to analyze the inter-observer reliability of both technologies among observers of various levels of skill experience.

## Materials and methods

### Subjects

Data were collected from May to July of the year 2022 in Jinling Hospital, Nanjing University School of Medicine. All procedures adhered to the tenets of the Declaration of Helsinki. The study was reviewed and approved by the Ethics Committee of the Jinling Hospital, Nanjing University School of Medicine(2022DZKY-041–01). Each participant signed a written informed consent after being notified of the purpose of the study and possible influence on the human body due to the exam.

This research involved 58 volunteers without any known ocular disease by self-report. Volunteers were recruited from a hospital and medical school campus; eligible volunteers underwent a series of routine eye examinations, and patients with eye conditions other than dry eye were excluded. In total, volunteers (25 males and 33 females) were qualified following by the inclusion and exclusion rules.

For each participant, we all choose their two eyes for measurement and statistical analysis.

### Diagnostic instruments

**Fig. 1:**
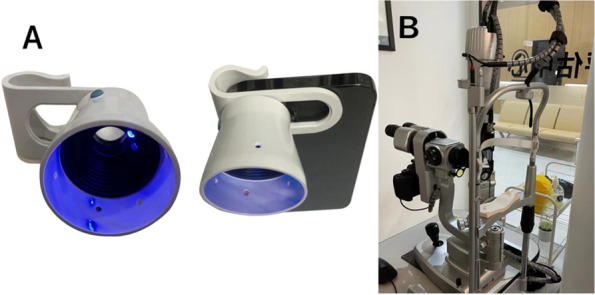
**A** The photo of the portable lens attachment for smartphones. **B** The photo of the conventional slit-lamp microscope

The LAS is an easily installed medical tool designed as seating on the camera of a cellphone (Patent CN 208,598,369; Fig. 1A). The LAS consists of a tube and an adjusted tube fixture. A convex macro lens (focal length: 40 mm, magnification: 4) in the tube is used to adjust the focus, and a light lamp is mounted at a 45-degree angle to strengthen the scattering effect of the light, which can emit blue light with a wavelength of 460–465 nm. Additionally, the lens is placed to be at a position that is 4 cm from the corneal apex, i.e. the focusing point for the convex lens. Furthermore, since the test for tear film breakage requires maintaining airflow, we modified this device by drilling eight holes around the tube to ensure proper airflow and TFBUT accuracy.

The iPhone 12 Pro (Apple Inc, Cupertino, CA, USA) was used for video recordings. The resolution of the video was 1080p, when the frame rate was 30 frames per second.

The recordable slit-lamp microscope (SL BQ900, RUIYU, China Fig. 1B) was used in this study for comparison as a conventional diagnostic method. Videos were recorded by the external camera fixed on the slit-lamp microscopes (Canon, Japan).

### Study design

The TFBUT test was taken on the same day using the slit-lamp microscopes and the LAS respectively, by an ophthalmologist with clinical expertise of more than 20 years. For the TFBUT examination, a drop of 50μL normal saline was placed on a fluorescence strip. The strip was then shaken to remove extra liquid from the stripe to minimize the interference with tear film volume. After that, the strip was placed to touch the inferior temporal bulbar conjunctiva of an eye gently. The subject was asked to blink several times to distribute the fluorescein over the cornea evenly.

Next, the subject was requested to keep the eyes open for as long as possible without blinking. The eye was then examined with three consecutive measurements using both the LAS and the slit-lamp microscopes for the TFBUT, both recorded with the video. The videos were stored on a computer in each session, and were then imported into the database for analysis. The calculated average TFBUT in three measurements was calculated and used for subsequent statistical analysis.

When TFBUT is equal to or shorter than 5 s, it was defined as tear film instability [9]. However, TFBUT values might be affected by subjects' compliance (partial vs. complete blinking), the skill level of practitioners, and environmental factors [15–18]. The TFBUT test was taken at 9:00 a.m. by slit lamp and at 12:00 a.m. by LAS in the same day, with the temperature (20–23 °C), and humidity (50–60%) kept constant [19].

Subsequently, an experienced ophthalmologist used a stopwatch to review all the videos stored in the database. Volunteers’ information was masked to avoid observation bias. Then two other observers of different training levels analyzed the same videos in the database (one postgraduate student, and one undergraduate student).

### Statistical and data analysis

The statistical analyses were performed with software SPSS for Windows, version 25 (International Business Machines Corporation, Armonk, NY, USA) and MedCalc, version 20.1 (Inc., Mariakerke, Belgium). The binocular data were used separately. We compared the TFBUT findings of two devices derived by the ophthalmologist using Spearman’s correlation coefficient and intraclass correlation coefficient (ICC). The Bland–Altman plot was used for visualization of the agreement between instruments. Then, the ICC was determined to compare TFBUT data obtained from the three different observers. A two-way random model accounts for potential systematic variations between observers. A *p*-value < 0.05 was considered statistically significant.

## Results

**Fig. 2:**
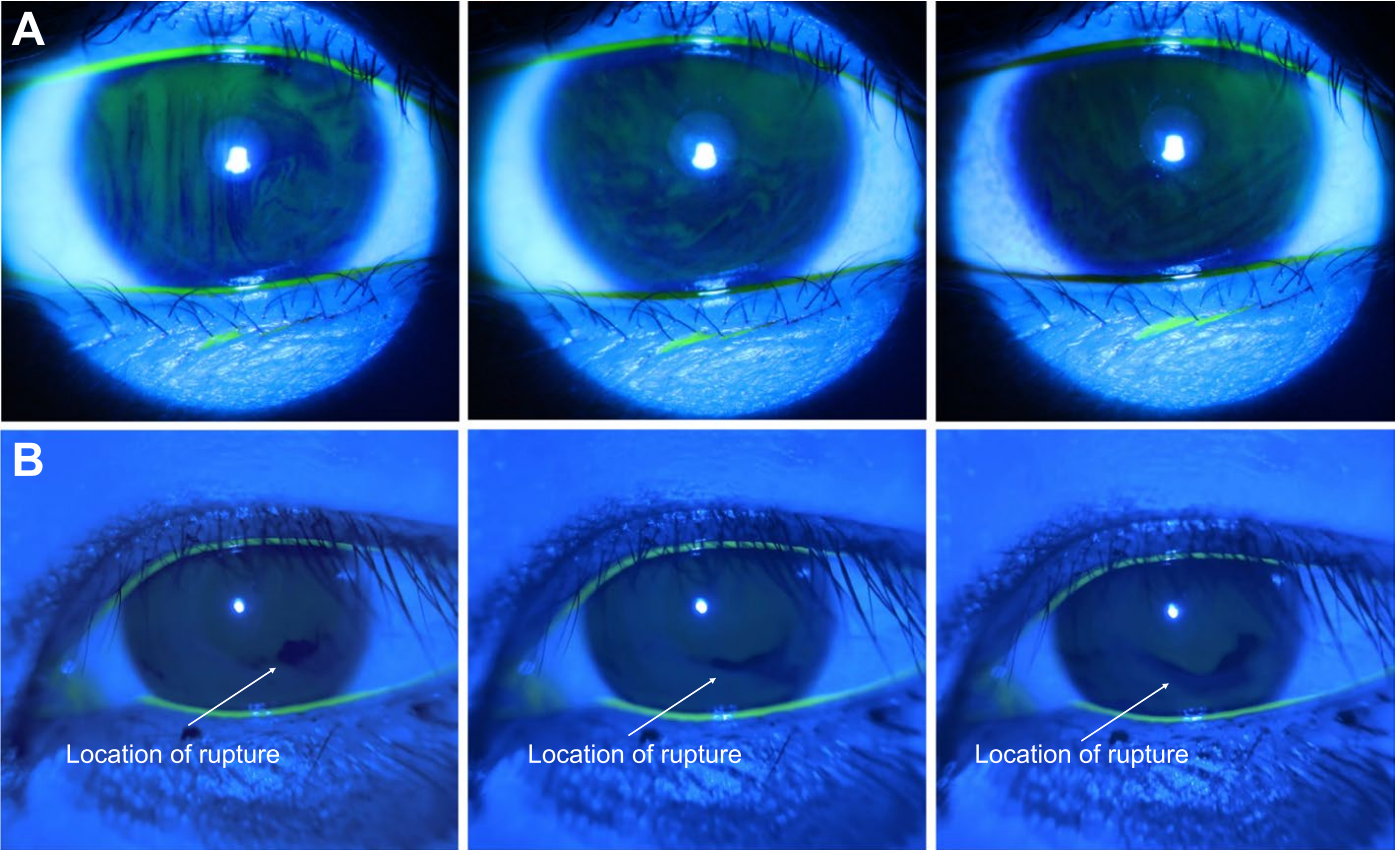
**A** The images were taken by the conventional recordable slit-lamp microscope. **B** The images were taken by the LAS. (Both images were from the same volunteer’s eyes)

A total of 116 eyes were included in the statistical analysis. The age of the volunteers ranged from 22 to 45 years old (mean age 28). A comparison of the optical properties between the slit-lamp microscope and the LAS is presented in Fig. 2.

**Fig. 3:**
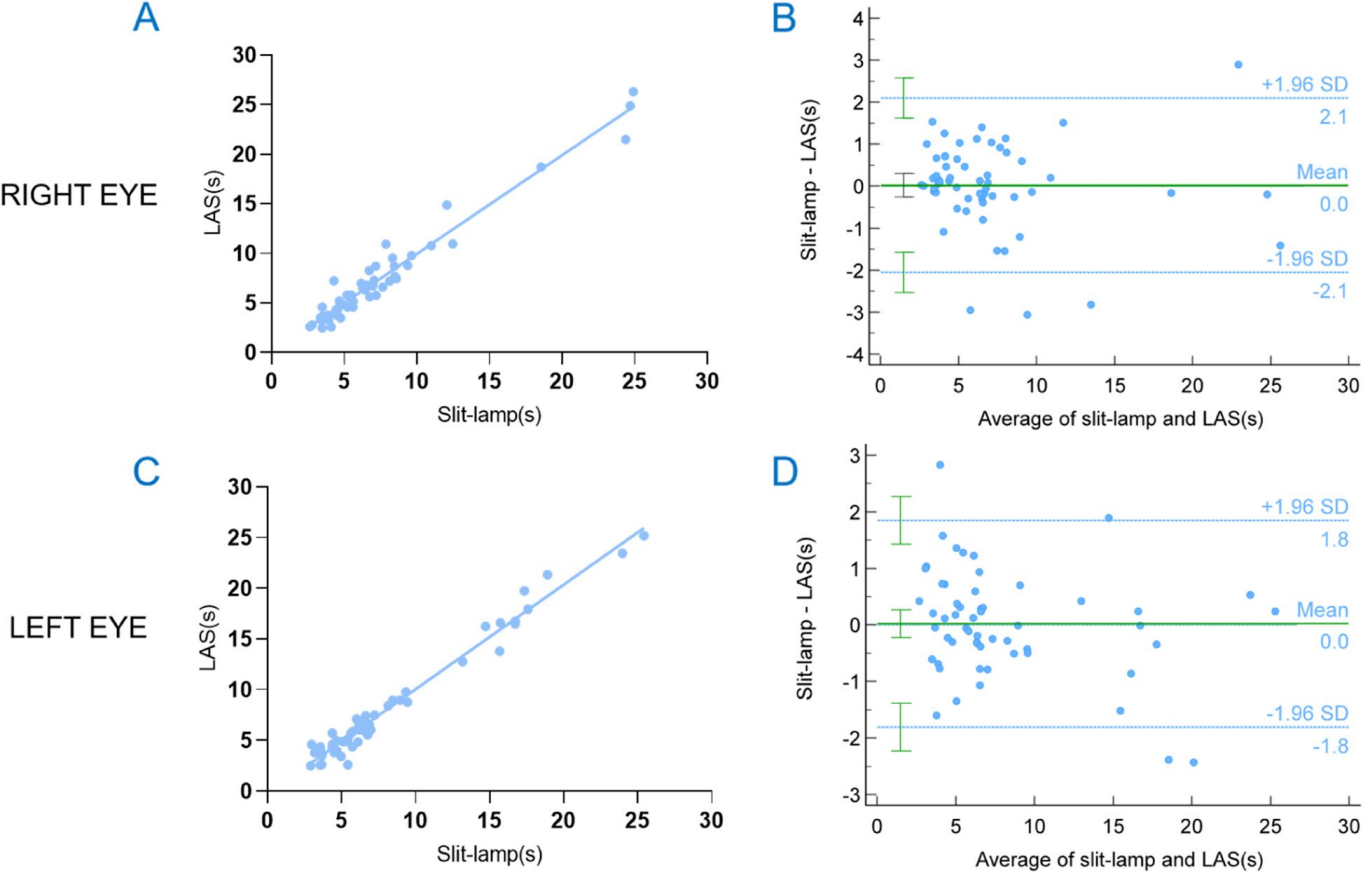
Correlation between slit-lamp microscopy and lens attachment for smartphones (LAS) The Bland–Altman plots show an agreement of TFBUT measurements between slit-lamp microscopy and LAS. The dots of right figures represent the differences from the mean value, the continuous line illustrates the mean value, and 95% limits of agreement (LoA) is also calculated as mean ± 1.96 standard deviation (SD) of the difference

We performed Spearman’s correlation and ICC for deciding the level of relevance between two modalities. There was a strong correlation between the conventional slit-lamp microscope and LAS regarding the TFBUT of the eye (Right eye: Spearman’s *r* = 0.929, 95% CI = 0.847–0.963, ICC = 0.978, *p* < 0.001; Left eye: Spearman’s *r* = 0.931, 95% CI = 0.866–0.964, ICC = 0.985, *p* < 0.001;) (Tables 1 and 2; Fig. 3). The Bland–Altman analysis showed good agreement between two devices (Fig. 3).

**Table 1:** Correlation between slit-lamp microscopy and LAS

	**n**	**R**^**a**^	**95% CI**
**TFBUT**			
**Right eye**	58	0.929	0.847–0.963
**Left eye**	58	0.931	0.866–0.964

*LAS* lens attachment for smartphones, *TFBUT* tear film break-up time, *CI* confidence interval

^a^Spearman’s rank correlation coefficient

**Table 2:** Inter-class correlation matrix between slit-lamp microscopy and LAS

	**LAS**	**95% CI**	**Sig**
**Right eye**			
Slit-lamp microscopy	0.978	0.962–0.987	< 0.001
**Left eye**			
Slit-lamp microscopy	0.985	0.975–0.991	< 0.001

1.000 represents a complete agreement, and lower numbers indicate less agreement

Table 3 showed ICC for LAS measurements of left eyes between an undergraduate student and a postgraduate student was 0.856, between an undergraduate student and an ophthalmologist was 0.929, between a postgraduate student and an ophthalmologist was 0.951, which suggested considerable consistency among observers.

**Table 3:** Inter-class correlation matrix regarding TFBUT calculated on the video obtained by LAS

	**Undergraduate Student**	**Postgraduate Student**	**Ophthalmologist**
**Right eye**			
Undergraduate Student	1.000	0.873	0.888
Postgraduate Student	0.873	1.000	0.944
Ophthalmologist	0.888	0.944	1.000
**Left eye**			
Undergraduate Student	1.000	0.856	0.929
Postgraduate Student	0.856	1.000	0.951
Ophthalmologist	0.929	0.951	1.000

1.000 represents a complete agreement, and lower numbers indicate less agreement

Table 4 showed ICC for silt-lamp microscopy measurements of left eyes between an undergraduate student and a postgraduate student was 0.860, between an undergraduate student and an ophthalmologist was 0.898, between a postgraduate student and an ophthalmologist was 0.944, which suggested considerable consistency among observers.

**Table 4:** Inter-class correlation matrix regarding TFBUT calculated on the video obtained by silt-lamp microscopy

	**Undergraduate Student**	**Postgraduate Student**	**Ophthalmologist**
**Right eye**			
Undergraduate Student	1.000	0.843	0.907
Postgraduate Student	0.843	1.000	0.936
Ophthalmologist	0.907	0.936	1.000
**Left eye**			
Undergraduate Student	1.000	0.860	0.898
Postgraduate Student	0.860	1.000	0.944
Ophthalmologist	0.898	0.944	1.000

1.000 represents a complete agreement, and lower numbers indicate less agreement

## Discussion

This is the first time that the LAS has been used for ophthalmology technology. In this study, we demonstrated the ability of the LAS for TFBUT measurement through objective findings in the clinical setting. When we compared the TFBUT measured between the conventional slit-lamp microscope and the LAS, a significantly strong correlation was found. These results suggest that the performance of the LAS is equivalent to the traditional slit-lamp microscope in assessing TFBUT.

Inter-observers reliability analysis indicated that this procedure could be carried out with excellent reproducibility. Our operators stood at various clinical experience levels, from novice (student) to advanced (ophthalmologist), meaning that this device can be used by individuals understanding medical knowledge at various extent. We suggest to further perfect the LAS with the eventual goal of spreading assessment of the TFBUT to places where silt-lamp microscopes are not available.

In the preliminary stage, this smartphone attachment still asks for the support of clinical novices, such as ophthalmic technicians, nurses, or individuals with relevant clinical training. To further demonstrate the feasibility of this technology, we asked three undergraduate students to measure each other’s TFBUT using this fresh tool at the end of the experiment. All three students successfully assessed the TFBUT and rated this product extremely favorably. We aim to develop a mobile app based on the image analysis technique, and the examiner simply captures tear film movie, and a TFBUT result would be offered as output.

In daily clinic, the slit-lamp microscope is a necessary medical equipment in the ophthalmic field. Nevertheless, most currently existing slit-lamp microscopes are costly and carry no recording function. Doctors are required to get an accurate diagnosis on site. However, as we all know, video recording of the examination can not only improve the accuracy of diagnosis and treatment, improve better communication between patient and doctors, but also increase the convenience for medical record retrievement. The accuracy of the diagnosis is better supported by videos or photos during the examination. In addition, the clinical examination videos can be stored in medical records, allowing repeat accessibility, which facilitates diagnosis and treatment. That’s why new tools are seriously needed for improving the efficiency and accessibility of both the clinical assessment and self-monitoring for DED.

At present, few devices capable of evaluating the ocular surface by attaching to a smartphone camera are available, although analogous technologies have previously been described. Yazu, et al. developed the "Smart Eye Camera (SEC)," a smartphone-based anterior segment examining device, and demonstrated its diagnostic effectiveness for DED. The device was registered as a medical device in Japan (Japan Medical Device registration number 13B2 10198030101) [12, 13, 20]. Compared with the SEC, our LAS has more advantages: (1) it can use the light source of the attached cellphone for blue light, with no need for additional light support; (2) the lens is designed to have a fixed distance of 4 cm off the cornea, which is another benefit of LAS. These make our products require a low level of clinical experience of the operator and have better popularity. Further analysis of other similar devices, the LAS may have potential advantages in medical care: First, the COVID-19 epidemic has driven numerous doctors and patients to embrace telemedicine [21–23]. Several research investigations have showed the reliability of telemedicine for diagnostics of certain ophthalmic diseases [21, 23]. Because the LAS is a portable device which is easy to operate, it is user-friendly for laymen people. Patients may use the LAS to test TFBUT at home, which increases the availability and compliance for basic DED testing [24]. The device may not only play an important role in telemedicine; which will mitigate overcrowding of hospitals, reduce population contact, and prevent viral transmission during the COVID-19 epidemic, but also be time-saving and economical for patients. Secondly, the LAS may be useful for evaluation of other ocular surface diseases. Several reports indicate the possibility of using smartphones for diagnosing another eye disease, such as cataract [13], conjunctival disorders [25], and narrow angles [14].

However, the LAS device also has some limitations. In the future, we intend to explore the usage of LAS in other ocular surface disease, the target group of this diagnostic tool. We are going to explore the feasibility of LAS in other ocular surface problems in subsequent study for better strength of our study about this novel diagnostic tool.

To conclude, using the smartphone with a combination of the LAS, TFBUT can be evaluated effectually. Although further study is needed, there is a strong hope that the results of this study could influence the further use of the LAS for diagnosing DED and ocular diseases.

## Data Availability

The datasets used and/or analyzed during the current study are available from the corresponding author on reasonable request.

## References

[1] Tsubota K, Pflugfelder SC, Liu Z (2020). Defining dry eye from a clinical perspective. Int J Mol Sci.

[2] Abelson MB, Ousler GW 3rd, Nally LA, et al. Alternative reference values for tear film break up time in normal and dry eye populations. Adv Exp Med Biol. 2002;506(Pt B):1121–5. 10.1007/978-1-4615-0717-8_157 12614039

[3] Bron AJ, de Paiva CS, Chauhan SK (2017). TFOS DEWS II pathophysiology report. Ocul Surf.

[4] Wu Y, Wang C, Wang X (2021). Advances in dry eye disease examination techniques. Front Med (Lausanne).

[5] Rouen PA, White ML (2018). Dry eye disease: prevalence, assessment, and management. Home Healthc.

[6] Noland ST, Badian RA, Utheim TP (2021). Sex and age differences in symptoms and signs of dry eye disease in a Norwegian cohort of patients. Ocul Surf.

[7] Willcox MDP, Argueso P, Georgiev GA (2017). TFOS DEWS II Tear Film Report. Ocul Surf.

[8] Wolffsohn JS, Arita R, Chalmers R (2017). TFOS DEWS II Diagnostic Methodology report. Ocul Surf.

[9] Craig JP, Nichols KK, Akpek EK (2017). TFOS DEWS II Definition and Classification Report. Ocul Surf.

[10] Norn MS (1969). Desiccation of the precorneal film. Acta Ophthalmol.

[11] Yazdani M, Fiskadal J, Chen X (2021). Tear film break-up time and dry eye disease severity in a Large Norwegian Cohort. J Clin Med.

[12] Shimizu E, Yazu H, Aketa N (2021). Smart eye camera: a validation study for evaluating the tear film breakup time in human subjects. Transl Vis Sci Technol.

[13] Yazu H, Shimizu E, Okuyama S (2020). evaluation of nuclear cataract with smartphone-attachable slit-lamp device. Diagnostics (Basel).

[14] Shimizu E, Yazu H, Aketa N (2021). A study validating the estimation of anterior chamber depth and iridocorneal angle with portable and non-portable slit-lamp microscopy. Sensors (Basel).

[15] Johnson ME, Murphy PJ (2005). The effect of instilled fuorescein solution volume on the values and repeatability of TBUT measure ments. Cornea.

[16] Paugh JR, Tse J, Nguyen T (2020). Efficacy of the fluorescein tear breakup time test in dry eye. Cornea.

[17] Mengher LS, Bron AJ, Tonge SR (1985). Effect of fluorescein instillation on the pre-corneal tear film stability. Curr Eye Res.

[18] Itokawa T, Okajima Y, Suzuki T (2018). Association between ocular surface temperature and tear film stability in soft contact lens wearers. Invest Ophthalmol Vis Sci.

[19] Pena-Verdeal H, Garcia-Resua C, Ramos L (2016). Diurnal variations in tear film break-up time determined in healthy subjects by software-assisted interpretation of tear film video recordings. Clin Exp Optom.

[20] Shimizu E, Ogawa Y, Yazu H (2019). "Smart Eye Camera": an innovative technique to evaluate tear film breakup time in a murine dry eye disease model. PLoS One.

[21] Parikh D, Armstrong G, Liou V (2020). Advances in Telemedicine in Ophthalmology. Semin Ophthalmol.

[22] Hallak JA, Scanzera AC, Azar DT (2020). Artificial intelligence in ophthalmology during COVID-19 and in the post COVID-19 era. Curr Opin Ophthalmol.

[23] Collon S, Chang D, Tabin G (2020). Utility and feasibility of teleophthalmology using a smartphone-based ophthalmic camera in screening camps in Nepal. Asia Pac J Ophthalmol (Phila).

[24] Sharma M, Jain N, Ranganathan S (2020). Tele-ophthalmology: need of the hour. Indian J Ophthalmol.

[25] Yazu H, Shimizu E, Sato S (2021). Clinical Observation of Allergic Conjunctival Diseases with Portable and Recordable Slit-Lamp Device. Diagnostics (Basel).

